# Supplementary material for: Identifying Early Metabolic Risks of Obesity in Mexican Children and Adolescents from a Semi-Rural Community in Mexico: Beyond BMI and into Biochemical and Nutritional Markers
Source: Nutrients. 2025 Jun 30;17(13):2195. doi: 10.3390/nu17132195 (PMC12251620; doi:10.3390/nu17132195)
Supplement: Supplementary file 1 [file nutrients-17-02195-s001.zip › nutrients-3701171-supplementary.pdf]

Supplementary Material

Supplementary Table S1. Recommended Dietary Allowances

Nutrient	RDA girls 4-8 yo	RDA girls 9-13 yo	RDA boys 4-8 yo	RDA boys 9-13 yo
Energy (kcal)	4-5 years – 1250<br>5-6 years – 1325<br>6-7 years – 1425<br>7-8 years – 1550<br>8-9 years – 1700	9-10 years – 1850<br>10-11 years – 2000<br>11-12 years – 2150<br>12-13 years – 2275	4-5 years – 1350<br>5-6 years – 1475<br>6-7 years – 1575<br>7-8 years – 1700<br>8-9 years – 1825	9-10 years – 1975<br>10-11 years – 2150<br>11-12 years – 2350<br>12-13 years – 2550
Protein (g/kg/day)	1.0	0.95	1.0	1.0
Percentage of energy as Protein	12-15	12-15	12-15	12-15
Fat	25-30	25-30	25-30	25-30
Percentage of energy as Fat	25-30	25-30	25-30	25-30
Carbohydrates (g/day)	130	130	130	130
Percentage of energy as Carbohydrates	55-63	55-63	55-63	55-63
Fiber (g/day)	18	22	18	22
Sugars	<5% from TEI	<5% from TEI	<5% from TEI	<5% from TEI
Cholesterol (mg/1000 kcal)	120-130	120-130	120-130	120-130
OMEGA 6 g/day	10 or<br>5-8% from TEI	10 or<br>5-8% from TEI	10 or<br>5-8% from TEI	12 or<br>5-8% from TEI
OMEGA 3 g/day	0.9<br>1-2 % from TEI	1.0<br>1-2 % from TEI	0.9<br>1-2 % from TEI	1.2<br>1-2% from TEI
Vitamin A µgER	400	590	400	580
Vitamin C (mg)	25	45	25	45
Vitamin D µg	5	5	5	5
Vitamin E (mg)	7	11	7	11
Vitamin K µg	55	60	55	60
Tiamin (mg)	0.5	0.8	0.5	0.8
Riboflavin (mg)	0.5	0.8	0.5	0.8
Niacin (mg)	8	12	8	12

Vitamin B6 (mg)	0.5	0.8	0.5	0.8
Folate	230	360	230	360
Vitamin B12 μg	1.2	1.7	1.2	1.7
Pantothenic acid (mg)	3.0	4.0	3.0	4.0
Isoleucine (mg/kg)	23 (0.23)	22 (0.22)	23 (0.23)	22 (0.22)
Leucine (mg/kg)	44 (0.044)	44 (0.044)	44 (0.044)	44 (0.044)
Lysine (mg/kg)	35 (0.035)	35 (0.035)	35 (0.035)	35 (0.035)
Methionine (mg/kg)	18 (0.018)	17 (0.017)	18 (0.018)	17 (0.017)
Phenylalanine (mg/kg)	30 (0.030)	30 (0.030)	30 (0.030)	30 (0.030)
Threonine (mg/kg)	18 (0.018)	18 (0.018)	18 (0.018)	18 (0.018)
Tryptophan (mg/kg)	4.8 (0.0048)	4.8 (0.0048)	4.8 (0.0048)	4.8 (0.0048)
Valine (mg/kg)	29 (0.029)	29 (0.029)	29 (0.029)	29 (0.029)
Cystina (mg/kg)	18 (0.018)	17 (0.017)	18 (0.018)	17 (0.017)
Cystine (mg/kg)	30 (0.030)	30 (0.030)	30 (0.030)	30 (0.030)
Tyrosine (mg/kg)	30 (0.030)	30 (0.030)	30 (0.030)	30 (0.030)
Calcium (mg)	800	1300	800	1300
Copper μg	440 (0.44)	700 (0.70)	440 (0.44)	680 (0.68)
Iron (mg)	15	16	15	20
Magnesium (mg)	130	240	130	240
Manganese mg/d (Upper intake level)	3	6	3	6
Phosphorus (mg)	500	1250	500	1250
Selenium μg	30	35	30	35
Potassium (mg/d)	1100	3100	1100	3100
Zinc (mg)	6.6	11.6	6.6	11.6
Sodium (mg/d)	700	1600	700	1600
Iodine μg	65	72	65	73

Abbreviations; RDA: Recommended Dietary Allowance, g: gram, gkcal: grams per kilocalorie, kcal: kilocalories, kg: kilogram, μg: microgram, μgER: micrograms of Retinol Equivalents, mg: milligram, TEI: Total Energy Intake, Vit: vitamin, yo: years old

Supplementary Table S2. Biochemical reference values

**Parameter**	**Reference value**
Glucose	60-100 mg/dL
Cholesterol	Reference <170<br>Limit 170-199<br>High >200
Triglycerides	Normal <150<br>Borderline high 150-199<br>High 200-499<br>Very high >500
High Density Lipoprotein (HDL)	Risk factor <40<br>Negative risk ≥ 60
Low Density Lipoprotein (LDL)	Optimal <100<br>High 160-189<br>Very high >190
Uric acid	2.40-5.50 mg/dL
AST	11-34 U/L
ALT	0-45 U/L
Interleukin 6	<10 pg/ml
Insulin	5-30 U/ml
GGT	12-64 U/L
Hematocrit	37-48%
Erythrocyte	4.30 - 5.66
Hemoglobin	13-17 g/dL
Total leucocyte count	3.5 - 9.080
Mean Corpuscular Volume	77-93 fL
Creatinine	0.52-0.69 mg/dL

Abbreviations: ALT: alanine aminotransferase, AST: aspartate aminotransferase, fL: femtoliter, g/dL: grams per deciliter, GGT: gamma-glutamyl transferase, mg/dL: milligrams per deciliter, pg/mL: picograms per milliliter, U/L: units per liter, U/mL: units per milliliter.

Supplementary Table S3. Total ingestion and differences in adequacy percentage of nutrients between children and adolescents.

	**Children**	**Adolescents**	**p Value**
Energy (kcal)	1254.1 ± 214.6	1147.2 ± 251.0	0.111
AP Energy	78.0 ± 14.1	55.4 ± 11.9	<0.0001
		**Macronutrients**	
Protein (g)	51.9 ± 13.7	48.9 ± 9.1	0.418
RDA Protein	23.4 ± 23.4	34.0 ± 7.9	<0.0001

AP Protein	226.9 ± 67.6	148.2 ± 33.2	<0.0001
Fat (g)	47.2 ± 10.9	44.2 ± 10.4	0.340
AP Fat (Maximum intake)	188.8 ± 43.5	176.7 ± 41.8	0.340
AP Fat (Minimum intake)	157.3 ± 36.3	147.3 ± 34.8	0.340
Carbohydrates (g)	156.8 ± 35.2	138.9 ± 37.8	0.095
AP Carbohydrates	120.6 ± 27.1	106.9 ± 29.1	0.095
Fiber (g)	10.1 ± 5.6	7.8 ± 3.3	0.120
AP Fiber	56.0 ± 31.2	35.3 ± 14.9	0.012
Sugar (g)	40.9 ± 20.6	34.1 ± 17.7	0.241
5% of the TEI	61.9 (55.2-69.9)	56 (49-68)	0.117
RDA Sugar	15.4 (13.8-17.4)	14 (12.2-17)	0.117
AP Sugar	247 (178-339)	240 (152-301)	0.423
Cholesterol	175.7 ± 72.6	193.8 ± 88.2	0.427
Cholesterol value 1	150.5 ± 25.7	137.7 ± 30.1	0.111
Cholesterol value 2	163.0 ± 27.9	149.1 ± 32.6	0.111
AP Cholesterol value 1	116.3 ± 42.3	151.6 ± 85.0	0.044
AP Cholesterol value 2	107.4 ± 39.0	139.9 ± 78.5	0.044
Percentage energy as Protein	16.5 ± 3.0	17.4 ± 3.6	0.330
Percentage energy as Fat	33.9 ± 4.8	34.7 ± 2.9	0.542
Percentage as Carbohydrates	49.6 ± 4.8	48.1 ± 5.2	0.295
**Micronutrients**			
Calcium (mg)	616.9 ± 372.8	453.0 ± 215.6	0.046
AP Calcium	77.1 ± 46.6	34.8 ± 16.6	<0.0001
Copper (mg)	0.3 ± 0.1	0.3 ± 0.1	0.920
AP Copper	68.8 ± 27.4	44.2 ± 11.6	<0.0001
Iron (mg)	7.6 ± 5.7	5.5 ± 1.9	0.051
AP Iron	50.3 ± 37.8	31.8 ± 12.6	0.009
Magnesium (mg)	99.2 ± 38.7	103.0 ± 29.7	0.718
AP Magnesium	76.3 ± 29.8	42.9 ± 12.4	<0.0001
Manganese (mg)	0.7 ± 0.5	0.5 ± 0.2	0.243
AP Manganese	23.1 ± 16.6	9.1 ± 3.6	<0.0001
Phosphorus (mg)	488.8 ± 174.5	526.1 ± 170.4	0.464
AP Phosphorus	97.8 ± 34.9	42.1 ± 13.6	<0.0001
Potassium (mg)	607.2 ± 232.2	662.3 ± 173.6	0.387
AP Potassium	55.2 ± 21.1	21.4 ± 5.6	<0.0001
Zinc (mg)	5.3 ± 4.7	3.9 ± 1.9	0.237
AP Zinc	80.7 ± 71.3	33.7 ± 16.1	<0.0001
Selenium (μg)	35.6 ± 12.5	40.8 ± 14.1	0.173
AP Selenium	118.6 ± 41.6	116.6 ± 40.2	0.869
Sodium (mg)	992.5 ± 327.3	901.6 ± 320.7	0.343

AP Sodium	141.8 ± 46.8	56.4 ± 20.0	<0.0001
Vit C (mg)	21.6 ± 26.9	16.1 ± 13.7	0.434
AP Vit C	86.3 ± 107.7	35.8 ± 30.4	0.011
Vit B6	5.8 ± 23.4	0.7 ± 0.2	0.373
AP Vit B6	1166.0 ± 4678.6	87.7 ± 20.9	0.003
Vit B12 (μg)	1.3 ± 0.8	1.1 ± 0.6	0.337
AP Vit B12	106.5 ± 70.4	62.3 ± 33.8	0.003
Vit D	2.1 ± 1.4	1.9 ± 1.3	0.506
AP Vit D	42.6 ± 28.7	37.2 ± 25.8	0.506
Vit E	1.4 ± 1.5	1.1 ± 0.6	0.468
AP Vit E	19.4 ± 21.5	9.9 ± 5.1	0.013
Vit K	6.2 ± 5.7	8.1 ± 7.4	0.296
AP Vit K	11.3 ± 10.3	13.6 ± 12.4	0.482
Thiamin	4.8 ± 19.5	0.5 ± 0.2	0.372
AP Thiamin	951.4 ± 3897.6	60.0 ± 22.9	0.352
Riboflavin	1.9 ± 9.1	3.7 ± 13.6	0.561
AP Riboflavin	371.7 ± 1819.7	459.8 ± 1702.2	0.561
Niacin	9.7 ± 9.4	11.7 ± 12.8	0.500
AP Niacin	120.8 ± 116.9	97.8 ± 106.3	0.493
Vit A	133.9 ± 91.2	117.3 ± 88.8	0.532
AP Vit A	33.5 ± 22.8	20.0 ± 15.0	0.013
Folate	122.0 ± 117.3	73.1 ± 40.5	0.027
AP Folate	53.0 ± 51.0	20.3 ± 11.3	<0.0001
Alpha linoleic acid	0.1 ± 0.0	0.1 ± 0.1	0.045
OMEGA 3	0.05 (0.02 - 0.10)	0.10 (0.04 - 0.15)	0.80
AP OMEGA 3	5.8 (2.7 - 11.2)	9.5 (4.3 - 15.4)	0.259
OMEGA 6	0.69 (0.34 - 1.6)	1.2 (0.45 - 2)	0.259
AP OMEGA 6	6.9 (3.4 - 16.1)	10.6 (4.2 - 19.5)	0.344
Amino Acids			
RDA Isoleucine	0.5 ± 0.1	0.8 ± 0.2	<0.0001
RDA Leucine	1.0 ± 0.2	1.6 ± 0.4	<0.0001
RDA Leucine	192.1 ± 65.7	149.3 ± 44.3	0.018
RDA Lysine	0.8 ± 0.2	1.2 ± 0.3	<0.0001
RDA Methionine	0.4 ± 0.1	0.6 ± 0.1	<0.0001
RDA Phenylalanine	0.7 ± 0.1	1.1 ± 0.3	<0.0001
AP Phenylalanine	140.1 ± 47.4	107.8 ± 35.3	0.015
RDA Threonine	0.4 ± 0.1	0.6 ± 0.1	<0.0001
AP Threonine	211.2 ± 76.6	168.7 ± 57.9	0.046
RDA Tryptophan	0.1 ± 0.0	0.2 ± 0.0	<0.0001
AP Tryptophan	205.1 ± 70.7	158.3 ± 58.7	0.021

RDA Valine	0.7 ± 0.1	1.0 ± 0.2	<0.0001
AP Valine	169.8 ± 59.9	134.3 ± 42.5	0.032
RDA Cystine	0.4 ± 0.1	0.6 ± 0.1	<0.0001
AP Cystine	69.2 ± 22.8	56.5 ± 17.1	0.046
RDA Histidine	0.7 ± 0.1	1.1 ± 0.2	<0.0001
RDA Tyrosine	0.7 ± 0.1	1.1 ± 0.2	<0.0001
AP Tyrosine	114.7 ± 39.5	85.5 ± 24.1	0.007

Abbreviations; AP: Adequacy Percentage, RDA: Recommended Dietary Allowance, g: gram, kcal: kilocalories, μg: microgram, mg: milligram, TEI: Total Energy ingestion, Vit: vitamin.

Supplementary Table S4. Plasma amino acid levels in children and adolescents.

**AA**	**Children**			**Adolescents**		
	NW *n = 26*	*OW* *n = 12*	*p value*	*NW* *n = 7*	*OW* *n = 8*	*p value*
ARG	30.7 (26.4-37.4)	33.2 (30.5-36.5)	0.195	36.7 (29.1-43.1)	31.2 (26.6-37.5)	0.281
CIT	24.2 (20.2-28.8)	23.7 (20.6-27.9)	0.938	25.7 (22.9-27.2)	25.6 (21.6-27.8)	1
GLY	277 (254-336.3)	258.4 (229.4-270.6)	0.093	285.5 (270.2-329.22)	266.7 (201.8-328.1)	0.232
ALA	229.5 (190.9-256.5)	250.4 (225.5-298.4)	0.1	264.6 (212.2-304.2)	273.6 (207.1-298.1)	1
LEU	103.7 (90.9-124.3)	124.4 (117.5-134.2)	0.010	115.2 (108.7-127.4)	142.1 (107.3-162.8)	0.463
MET	16.2 (13.1-18.3)	17.9 (15.5-19)	0.155	17.6 (15.8-18.1)	17.5 (14.5-22.6)	0.955
PHE	37.4 (34.9-41.7)	44.2 (42.5-45-5)	0.016	40.1 (37.3-51.4)	50 (39.5-70.9)	0.463
TYR	53.8 (46.4-59.5)	74.5 (54.3-84.9)	0.025	54 (50.5-63.6)	62.7 (48.7-73.7)	0.463
VAL	140.2 (122.2-159.5)	177.1 (155.4-193.4)	0.001	145.5 (126-151.4)	203.3 (137.6-213.2)	0.281
ORN	61.7 (55.3-71.5)	67.6 (54.3-75.6)	0.653	64 (50.1-81.5)	67.7 (55.4-75.8)	1
PRO	123.3 (98.8-145.6)	156.2 (146.5-194.7)	0.003	138 (103-149.4)	175.2 (122.2-211.6)	0.397

Abbreviations; AA: amino acid, ALA: alanine, ARG: arginine, CIT: citrulline, GLY: glycine, LEU: leucine, MET: methionine, NW: normal weight, ORN: ornithine, OW: overweight, PHE: phenylalanine, PRO: proline, TYR: tyrosine, VAL: valine.
